# Metajapogenins A–C, Pregnane Steroids from Shells of *Metaplexis japonica*

**DOI:** 10.3390/molecules22040646

**Published:** 2017-04-17

**Authors:** Hui-Li Yao, Yang Liu, Xiao-Hong Liu, Hua Gao, Kun Liu, Yan-Lin Shao, Xin-Yu Fang, Wei Wang

**Affiliations:** School of Pharmacy, Qingdao University, Qingdao 266021, Shandong, China; xyhuili@126.com (H.-L.Y.); buckuper@163.com (Y.L.); liuxiaohong1043@163.com (X.-H.L.); gaohuaqy@126.com (H.G.); kunliu62@126.com (K.L.); yanlinlz@126.com (Y.-L.S.); fangxinyu318@163.com (X.-Y.F.)

**Keywords:** *Metaplexis japonica* (Thunb.) Makino, Apocynaceae, pregnane steroid, metajapogenins A–C

## Abstract

Phytochemical investigation of the shells of *Metaplexis japonica* (Thunb.) Makino, belonging to the family of Apocynaceae, afforded three new pregnane steroids, metajapogenins A–C, along with three known compounds. The structures of the new compounds were elucidated as 12β,14β,17β-trihydroxypregna-3,5-dien-7,20-dione, 12β,14β,17β,20β-tetrahydroxypregna-3,5-dien-7-one; 3β,12β,14β,17β-tetrahydroxypregn-5-ene-7,20-dione on the basis of extensive spectroscopic evidence derived from 1D; 2D-NMR experiments and mass spectrometry. The known compounds included pergularin; 12-*O*-acetylpergularin; and pergularin-3-*O*-β-d-oleandropyranose; which were identified for the first time in the shells of *M*. *japonica*.

## 1. Introduction

**Figure 1:**
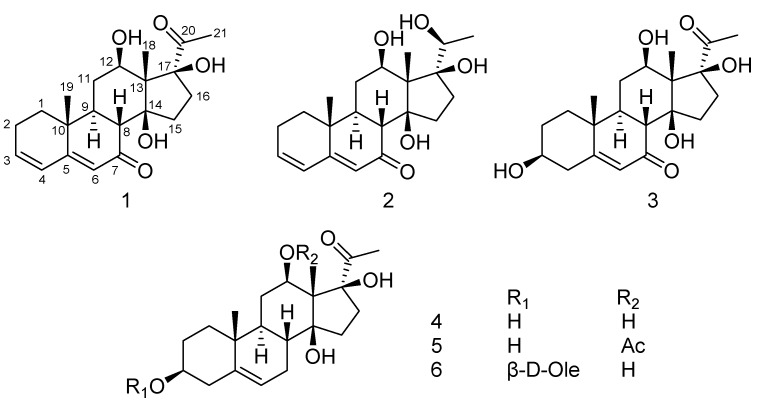
Structures of compounds **1**–**6**.

The genus *Metaplexis* (Apocynaceae family) consists of six species which are distributed throughout eastern Asia [1]. *Metaplexis japonica* (Thunb.) Makino is a climbing perennial herb with a comprehensive distribution in China, Japan, Korea, and adjacent Russia, and has been used as a traditional Chinese medicine in China. The stems and roots are used for the treatment of traumatic injury, snake bites, impotence, and infantile malnutrition due to intestinal parasites. The fruits are applied to cure weakness, cough, internal lesion caused by over exertion, and lumbar and leg pain [2]. Previous studies revealed that the major secondary metabolites present in the roots and aerial parts of *M*. *japonica* are pregnane steroids and flavonol glycosides [3,4,5,6,7,8,9,10]. Regarding the biological potential of *M*. *japonica*, previous studies have reported the antibacterial and antioxidant activities of the essential oils [11], antioxidant activity of the extract and derivatives [12,13], immunosuppressive activity of the purified total polysaccharides [14], and neuroprotective effects of the extract on global and focal cerebral ischemia in rat models [15]. However, little attention has been focused on the constituents in the shell of *M*. *japonica*. As part of our ongoing efforts to discover bioactive metabolites from the herbs of the Apocynaceae family [16,17,18], three new pregnane steroids, metajapogenins A–C, together with three known compounds, pergularin, 12-*O*-acetylpergularin, and pergularin-3-*O*-β-d-oleandropyranose, were isolated and identified from the shells of *M. japonica*. This paper describes the isolation and structure elucidation of these compounds (Figure 1).

## 2. Results and Discussion

**Figure 2:**
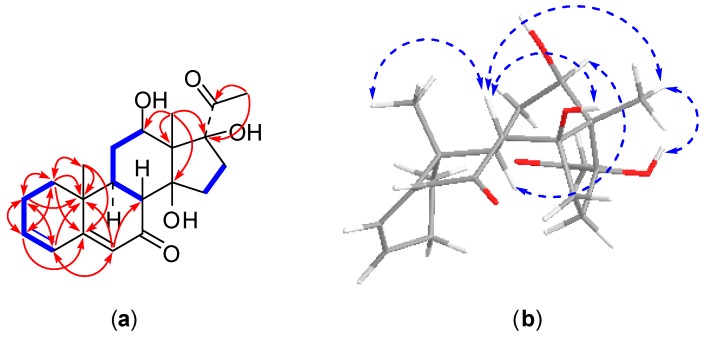
2D NMR correlations of compound **1**: (**a**) ^1^H-^1^H correlations (bold lines) and selected HMBC correlations (arrows); (**b**) selected NOSEY correlations.

Compound **1** was obtained as an amorphous solid. Its molecular formula was determined as C_21_H_28_O_5_ on the basis of positive HRESI-MS (*m*/*z* 361.1978 [M + H]^+^, calcd. for C_21_H_29_O_5_, 361.2015) and ^13^C-NMR data. The ^13^C-NMR spectrum displayed 21 carbon resonances involving three methyl groups at δ_C_ 7.8 (C-18), 16.5 (C-19), and 27.7 (C-21), five methylene carbons at δ_C_ 23.5 (C-2), 31.6 (C-15), 31.8 (C-11), 32.6 (C-1), and 33.6 (C-16), three methine carbons (one oxygenated) at δ_C_ 43.4 (C-9), 48.5 (C-8), and 67.2 (C-12), four quaternary carbons (two oxygenated) at δ_C_ 36.2 (C-10), 59.3 (C-13), 87.8 (C-14), and 91.7 (C-17), four olefinic carbons at δ_C_ 124.0 (C-6), 127.5 (C-4), 139.4 (C-3) and 164.2 (C-5), and two carbonyl carbons at δ_C_ 203.0 (C-7) and 209.1 (C-20) (Table 1), indicating compound **1** is a pregnane derivative, which is consistent with the ^1^H-NMR displaying characteristic signals for three methyl protons at δ_H_ 1.01 (3H, s, H-19), 1.70 (3H, s, H-18), and 2.62 (3H, s, H-21), one oxygenated methine proton at δ_H_ 3.78 (1H, dd, *J* = 11.4, 4.5 Hz, H-12), and three olefinic protons at δ_H_ 5.80 (1H, s, H-6), 6.09 (1H, dd, *J* = 9.8, 1.9 Hz, H-4), 6.16 (1H, m, H-3) (Table 2). Comprehensive analyses of the 2D-NMR spectra of compound **1** allowed us to establish its structure, as shown in Figure 1. The connectivity of the protonated carbons (C-1 to C-2, C2 to C-3, C-3 to C-4, C-8 to C-9, C-9 to C-11, C-11 to C-12, and C-15 to C-16) was determined from the ^1^H-^1^H COSY spectrum. In the HMBC spectrum, the methyl protons of H_3_-18 correlated to one oxygenated methine carbon at C-12, two oxygenated quaternary carbons at C-14 and C-17, and one quaternary carbon at C-13, which revealed the hydroxy groups are located at C-12, C-14 and C-17, respectively (Figure 2). The assignment of a conjugated double bond at the C-3 and C-5 was supported by the HMBC correlations from H-1 (δ_H_ 1.21 and 1.80) to C-2, C-10, C-3, C-5, and C-19, from H-2 (δ_H_ 2.03 and 2.15) to C-1, C-3, C-4, and C-10, from H-3 to C-2, C-1, and C-5, from H-4 to C-5, C-2, C-6, and C-10, from H-6 to C-4, C-8, and C-10, and from H-19 to C-10, C-1, C-5, and C-9. Furthermore, the HMBC correlations with the methyl protons of H_3_-21 to the carbonyl carbon C-20 and two oxygenated quaternary carbon of C-17 revealed the placement of the carbonyl carbon at C-20. The downfield shift of C-7 from about δ_C_ 27.0 [4] to δ_C_ 203.0 indicated the presence of an additional carbonyl carbon at C-7. The position of the C-7 carbonyl carbon was confirmed from the downfield shift at C-5, as well as from the singlet at δ_H_ 5.80 (H-6) [19]. Thus, the planer structure of compound **1** was established as 12,14,17-trihydroxypregna-3,5-dien-7,20-dione. The relative configuration of compound **1** was determined by analysis of vicinal proton-proton coupling and NOESY experiment (Figure 2). The large ^3^*J* coupling constant of H-8 and H-9 (*J* = 12.9 Hz) established the *trans*-diaxial orientation of H-8 and H-9 [20]. The NOE correlations from H-8 (δ_H_ 2.82) to both H_3_-18 and H_3_-19 indicated that these protons were β-oriented and that *trans*-fused geometry occurred at the ring junction [21]. The NOE correlations from H-8 to the hydroxy group at C-14 and from H_3_-18 to the hydroxy groups at C-14 and C-17 led to the assignment of β-orientation for the hydroxy groups at C-14 and C-17, respectively. In addition, the hydroxy group at C-12 was determined to be β-oriented based on the NOE cross peak between H-9 (δ_H_ 1.83) and H-12. Therefore, the structure of compound **1** was elucidated as 12β,14β,17β-trihydroxypregna-3,5-dien-7,20-dione and named metajapogenin A.

**Figure 3:**
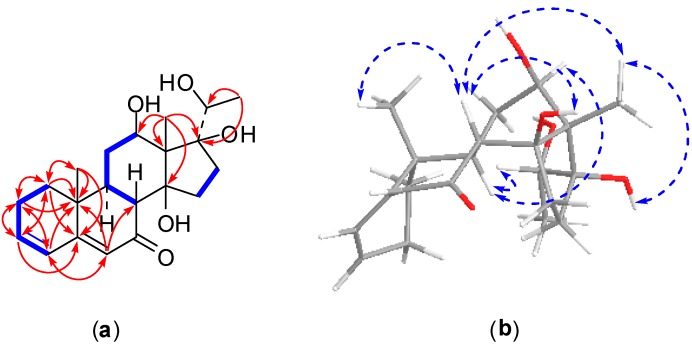
2D NMR correlations of compound **2**: (**a**) ^1^H-^1^H correlations (bold lines) and selected HMBC correlations (arrows); (**b**) selected NOSEY correlations.

Compound **2**, an amorphous powder, exhibited a molecular formula of C_21_H_30_O_5_ evidenced by the molecular ion peak [M + H]^+^ at *m*/*z* 363.2123 (calcd. for C_21_H_31_O_5_, 363.2172) with seven degrees of unsaturation found using HRESI-MS. The NMR data (Table 1 and Table 2) showed one oxygenated methine proton at δ_H_ 3.78 (1H, dd, *J* = 11.4, 4.5 Hz, H-12), which was relevant to oxygenated methine carbon at δ_C_ 69.4, and three olefinic protons at δ_H_ 5.80 (1H, s, H-6), 6.09 (1H, br d, *J* = 9.8 Hz, H-4), 6.16 (1H, m, H-3), which were relevant to olefin carbons at δ_C_ 123.7 (C-6), 127.5 (C-4), and 139.5 (C-3). Moreover, in the ^13^C-NMR spectrum, one nonprotonated olefinic carbon signal located at δ_C_ 164.3 (C-5), two oxygenated quaternary carbon resonances presented at δ_C_ 87.5 (C-14) and 87.6 (C-17), and one carbonyl carbon signal appeared at δ_C_ 203.4 (C-7). Careful analysis of the NMR spectra of compound **2** indicated that the structure of **2** was similar to that of compound **1**, except for in the vicinity of the side chain at C-17. The signal for the carbonyl group at δ_C_ 209.1 (C-20) in the ^13^C-NMR spectrum of compound **1** was replaced by an oxygenated methine carbon signal at δ_C_ 73.1 (C-20) in compound **2**. Meanwhile, resonance of the singlet methyl group at δ_H_ 2.62 (3H, s, H-21) in the ^1^H-NMR spectrum of compound **1** changed to a doublet methyl protons signal at δ_H_ 1.60 (3H, d, *J* = 6.4 Hz, H-21) in compound **2**. The HMBC correlations of H_3_-21 to C-17 and C-20 supported this deduction (Figure 3). The β-orientations for the hydroxy groups at C-12, C-14, and C-17 were consistent with those of compound **1** based on the detailed analysis of a NOESY spectrum (Figure 3). Furthermore, the hydroxy group at C-20 was determined to be β-oriented by the NOESY correlations from H-12 to H-9 and H-20. Thus, the structure of compound **2** was established as 12β,14β,17β,20β-tetrahydroxypregna-3,5-dien-7-one and was assigned a trivial name metajapogenin B.

**Figure 4:**
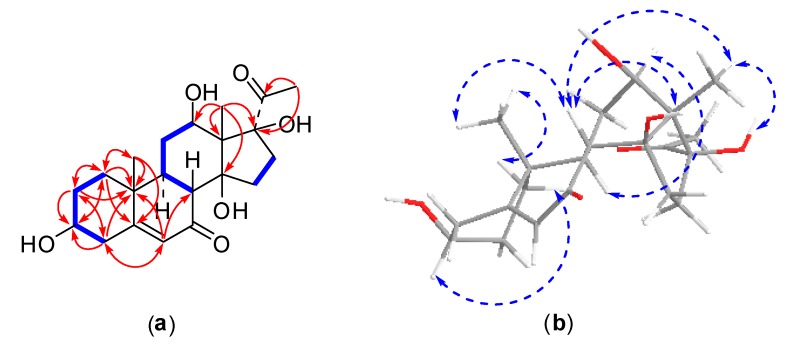
2D NMR correlations of compound **3**: (**a**) ^1^H-^1^H correlations (bold lines) and selected HMBC correlations (arrows); (**b**) selected NOSEY correlations.

Compound **3** was obtained as an amorphous powder. The HRESI-MS spectrum showed a positive molecular ion peak at *m*/*z* 379.2115 [M + H]^+^, corresponding to a molecular formula of C_21_H_30_O_6_ (calcd. for C_21_H_31_O_6_, 379.2121), which was further supported by the NMR spectral data. The ^13^C-NMR spectrum displayed 21 carbon signals (Table 1). Two carbonyl carbons located at δ_C_ 202.5 (C-7) and 209.1 (C-20), two olefinic carbons appeared at δ_C_ 126.0 (C-6) and 171.0 (C-5). Signals for two oxygenated methine carbons and two oxygenated quaternary carbons observed at δ_C_ 67.3 (C-12), 69.8 (C-3), 87.7 (C-14), and 91.7 (C-17). As judged from the DEPT and HSQC spectra, the remaining carbon resonances were three methyl carbons, six methylene carbons, two methine carbons, and two quaternary carbons. This spectral data of compound **3** was similar to compound **1** except for the replacements of two olefinic carbons at C-3 and C-4 with one oxygenated methine carbon and one methylene carbon, respectively. Those were confirmed by HMBC correlations of H-1 (δ_H_ 1.12)/C-3, H-2 (δ_H_ 1.78)/C-3, and H-4 (δ_H_ 2.60)/C-3 (Figure 4). Compound **3** showed very similar NOESY correlations to those of compound **1** (Figure 4). Moreover, the NOE correlations from H-1a (δ_H_ 1.86) to H_3_-19 (δ_H_ 1.12) and from H-1b (δ_H_ 1.17) to H-3 (δ_H_ 3.89) indicated an α-axial configuration of H-3 and β-orientation of the hydroxy group at C-3. On the basis of the above evidence, the structure of compound **3** was determined to be 3β,12β,14β,17β-tetrahydroxypregn-5-ene-7,20-dione and a trivial name metajapogenin C was given.

Additionally, the three known compounds were identified as pergularin (**4**) [7], 12-*O*-acetylpergularin (**5**) [3], and pergularin-3-*O*-β-d-oleandropyranose (**6**) [22] by comparison of their spectral data with those reported in the literature.

## 3. Materials and Methods

### 3.1. General Experimental Procedures

HR-ESI-MS and ESI-MS were obtained with a Bruker microTOFQ mass spectrometer (Bruker Daltonics, Bremen, Germany). The NMR spectral data were recorded on a Bruker AV-500 FT-NMR (500 MHz for ^1^H and 125 MHz for ^13^C) in C_5_D_5_N, using visual C_5_D_5_N resonances (δ_H_ 7.21, 7.58, and 8.73, δ_C_ 123.5, 135.5, and 149.0) for internal reference. All chemical shifts (δ) are given in ppm. Optical rotations were measured by using a JASCO P-1020 automatic digital polarimeter (JASCO Corporation, Tokyo, Japan). Preparative HPLC was performed on a NP7005C pump connected with a SHODEX RI-102 detector (Shoko Scientific, Tokohama, Japan), using Megres ODS column (250 mm × 20 mm, i.d., 5 μm, Hanbang Sci. and Tech., Haian, China). Column chromatography was performed with macroporous resin HPD100 (Cangzhou Bon Adsorber Technology, Cangzhou, China) and RP-18 reversed-phase silica gel (S-50 mm, YMC, Kyoto, Japan). TLC analysis was carried out on pre-coated TLC plates with silica gel RP-18 60 F_254_ (Merck, Darmstadt, Germany, 0.25 mm). Detection was achieved by spraying with 10% H_2_SO_4_ in MeOH followed by heating. HPLC-grade MeOH was purchased from Merck. HPLC-grade water was purified using a Milli-Q system (millipore, Boston, MA, USA). All solvents used for the chromatographic separations were distilled before use.

### 3.2. Plant Material

The shells of *Metaplexis japonica* (Thunb.) Makino were collected from Changbai Mountain, Jilin Province of China, in October 2012, and authenticated by Prof. Bomin Feng, College of Life Science and Technology, Dalian University, China. A voucher specimen (MJLMK20121001) was deposited at the College of Pharmacy, Qingdao University, China.

### 3.3. Extraction and Isolation

The dried and ground shells of *M*. *japonica* (9.5 Kg) were extracted with 90% aqueous EtOH to produce a crude extract (810 g). The crude extract was suspended in water and then filtered. The soluble fraction was subjected to column chromatography on D101 macroporous resin and eluted with 30%, 70%, and 90% aqueous EtOH, successively. The fractions eluted with 70% and 90% aqueous EtOH were chromatographed on a D941 macroporous resin column, eluted with 95% aqueous EtOH to give 3.3 g and 3.2 g residues, respectively. The residue eluted with 70% aqueous EtOH was isolated further on a RP-C_18_ silica gel and eluted with a gradient increasing MeOH (30–50%) in water to give sixteen subfractions (Fr. 70-1~Fr. 70-17) on the basic of TLC analyses. Fr. 70-16 was purified by preparative HPLC using MeOH/H_2_O (60:40) at a flow rate 2.0 mL/min resulting in the isolation of compound **1** (53.0 mg, *t*_R_ = 130 min). Compound **3** (17.3 mg, *t*_R_ = 190 min) and compound **4** (54.7 mg, *t*_R_ = 150 min) were obtained from Fr. 70-11 by preparative HPLC employing MeOH/H_2_O (25:75) as the mobile phase. Fr. 70-15 was chromatographed by preparative HPLC using MeOH/H_2_O (60:40) at a flow rate 2.0 mL/min to yield compound **5** (19.1 mg, *t*_R_ = 90 min). The residue eluted with 90% aqueous EtOH was separated chromatographically on a RP-C_18_ silica gel to afford seven subfractions (Fr. 90-1~Fr. 90-7) on the basis of TLC analysis. compound **2** (3.0 mg, *t*_R_ = 115 min) were obtained from Fr. 90-4 by preparative HPLC (flow rate, 2.0 mL/min) employing MeOH/H_2_O (60:40) as the mobile phase. Fr. 90-2 was isolated by preparative HPLC using MeOH/H_2_O (60:40) at a flow rate 2.0 mL/min to yield compound **6** (1.1 mg, *t*_R_ = 70 min).

Compound **1**: white amorphous power; $[\alpha {]}_{25}^{D}$ ‒32.6 (*c* 0.12, MeOH); HRESI-MS *m*/*z* 361.1978 [M + H]^+^ (calcd. for C_21_H_29_O_5_, 361.2015); ^1^H-NMR (C5D5N, 500 MHz) and ^13^C-NMR (C_5_D_5_N, 125 MHz) spectra data, see Table 1 and Table 2.

Compound **2**: white amorphous powder; $[\alpha {]}_{25}^{D}$ −53.2 (*c* 0.15, MeOH); HRESI-MS *m*/*z* 363.2123 [M + H]^+^ (calcd. for C_21_H_31_O_5_, 363.2172); ^1^H-NMR (C_5_D_5_N, 500 MHz) and ^13^C-NMR (C_5_D_5_N, 125 MHz) spectra data, see Table 1 and Table 2.

Compound **3**: white amorphous powder; $[\alpha {]}_{25}^{D}$ −24.9 (*c* 0.10, MeOH); HRESI-MS *m*/*z* 379.2115 [M + H]^+^ (calcd. for C_21_H_31_O_6_, 379.2121); ^1^H-NMR (C_5_D_5_N, 500 MHz) and ^13^C-NMR (C_5_D_5_N, 125 MHz) spectra data, see Table 1 and Table 2.

Compound **4**: white amorphous powder; ESI-MS *m*/*z* 365 [M + H]^+^; ^1^H-NMR (C_5_D_5_N, 500 MHz) δ: 1.18 and 1.87 (each 1H, m, H_2_-1), 1.80 and 2.10 (each 1H, m, H_2_-2), 3.87 (1H, m, H-3), 2.57 and 2.62 (each 1H, m, H_2_-4), 5.48 (1H, t, *J* = 2.6 Hz, H-6), 1.99 and 2.52 (each 1H, m, H_2_-7), 2.06 (1H, m, H-8), 1.32 (1H, ddd, *J* = 12.6, 12.4, 4.0 Hz, H-9), 1.96 and 2.06 (each 1H, m, H_2_-11), 3.81 (1H, m, H-12), 1.74 and 1.93 (each 1H, m, H_2_-15), 2.12 and 3.44 (each 1H, m, H_2_-16), 1.76 (3H, s, H_3_-18), 1.09 (3H, s, H_3_-19), 2.63 (3H, s, H_3_-21); ^13^C-NMR (C_5_D_5_N, 125 MHz) δ: 37.2 (t, C-1), 31.7 (t, C-2), 71.2 (d, C-3), 43.4 (t, C-4), 140.9 (s, C-5), 121.6 (d, C-6), 26.8 (t, C-7), 37.1 (d, C-8), 43.8 (d, C-9), 37.7 (s, C-10), 32.5 (t, C-11), 68.3 (d, C-12), 59.1 (s, C-13), 89.0 (s, C-14), 31.8 (t, C-15), 32.6 (t, C-16), 92.4 (s, C-17), 7.7 (q, C-18), 19.8 (q, C-19), 209.2 (s, C-20), 27.8 (q, C-21).

Compound **5**: white amorphous powder; ESI-MS *m*/*z* 407 [M + H]^+^; ^1^H-NMR (C_5_D_5_N, 500 MHz) δ: 1.10 and 1.78 (each 1H, m, H_2_-1), 1.74 and 2.15 (each 1H, m, H_2_-2), 3.83 (1H, m, H-3), 2.60 and 2.65 (each 1H, m, H_2_-4), 5.43 (1H, t, *J* = 2.6 Hz, H-6), 1.99 and 2.48 (each 1H, m, H_2_-7), 2.05 (1H, m, H-8), 1.40 (1H, ddd, *J* = 12.6, 12.4, 4.0 Hz, H-9), 1.96 and 2.02 (each 1H, m, H_2_-11), 4.87 (1H, dd, *J* = 11.5, 4.8 Hz, H-12), 1.63 and 1.94 (each 1H, m, H_2_-15), 2.08 and 3.31 (each 1H, m, H_2_-16), 1.66 (3H, s, H_3_-18), 1.04 (3H, s, H_3_-19), 2.50 (3H, s, H_3_-21), 2.08 (3H, s, H_3_-COCH_3_); ^13^C-NMR (C_5_D_5_N, 125 MHz) δ: 37.2 (t, C-1), 31.5 (t, C-2), 71.1 (d, C-3), 43.2 (d, C-4), 140.8 (s, C-5), 121.2 (d, C-6), 27.0 (t, C-7), 37.2 (d, C-8), 43.3 (d, C-9), 37.5 (s, C-10), 26.6 (t, C-11), 73.2 (d, C-12), 56.7 (s, C-13), 88.9 (s, C-14), 32.4 (t, C-15), 32.6 (t, C-16), 92.1 (s, C-17), 8.6 (q, C-18), 19.6 (q, C-19), 209.7 (s, C-20), 27.4 (q, C-21), 169.8 (C-COCH_3_), 20.7 (C-COCH_3_).

Compound **6**: white amorphous powder; ESI-MS *m*/*z* 509 [M + H]^+^; ^1^H-NMR (C_5_D_5_N, 500 MHz) δ: 1.12 and 1.82 (each 1H, m, H_2_-1), 1.80 and 2.15 (each 1H, m, H_2_-2), 3.86 (1H, m, H-3), 2.45 and 2.63 (each 1H, m, H_2_-4), 5.53 (1H, t, *J* = 2.6 Hz, H-6), 2.00 and 2.52 (each 1H, m, H_2_-7), 2.04 (1H, m, H-8), 1.32 (1H, m, H-9), 1.92 and 2.08 (each 1H, m, H_2_-11), 3.82 (1H, m, H-12), 1.75 and 1.93(1H, m, H_2_-15), 2.13 and 2.43 (1H, m, H_2_-16), 1.77 (3H, s, H_3_-18), 1.05 (3H, s, H_3_-19), 2.66 (3H, s, H_3_-21), 4.89 (1H, dd, *J* = 9.9, 1.8 Hz, H-1′), 1.82 and 2.56 (each 1H, m, H_2_-2′), 3.52 (1H, m, H-3′), 3.49 (1H, m, H-4′), 3.61 (1H, m, H-5′), 1.60 (3H, d, *J* = 6.0 Hz, H-6′), 3.47 (3H, s, H_3_-OCH_3_); ^13^C-NMR (C_5_D_5_N, 125 MHz) δ: 37.3 (t, C-1), 31.6 (t, C-2), 72.9 (d, C-3), 39.3 (t, C-4), 140.0 (s, C-5), 122.6 (d, C-6), 26.8 (t, C-7), 36.99 (d, C-8), 43.77 (d, C-9), 37.40 (s, C-10), 31.60 (t, C-11), 68.21 (d, C-12), 59.02 (s, C-13), 88.87 (s, C-14), 30.3 (t, C-15), 31.7 (t, C-16), 92.4 (s, C-17), 7.6 (q, C-18), 19.6 (q, C-19), 209.0 (s, C-20), 27.7 (q, C-21), 98.2 (d, C-1′), 37.5 (t, C-2′), 81.7 (d, C-3′), 77.4 (d, C-4′), 72.9 (d, C-5′), 18.8 (q, C-6′), 57.0 (C-OCH_3_).

## 4. Conclusions

In this study, three new pregnane steroids, metajapogenins A–C, together with three known compounds, pergularin, 12-*O*-acetylpergularin, and pergularin-3-*O*-β-d-oleandropyranose, were isolated and identified from the shells of *M. japonica*. To our best knowledge, metajapogenins A and B are the first examples of naturally occurring pregna-3,5-dien-7-one steroid. Pergularin and 12-*O*-acetylpergularin were only isolated from the root of *M*. *japonica* while pergularin-3-*O*-β-d-oleandropyranose was reported from the aerial part of *Cynanchum formosanum*. The known compounds were found for the first time in the shell of *M*. *japonica*. The isolation of six compounds from the shell of *M*. *japonica* is the first phytochemistry study and may be used as a foundation for further chemotaxonomic studies on the genus *Metaplexis*.

## Figures and Tables

**Table 1:** ^13^C-NMR spectral data of compounds **1**–**3** (125 MHz, C_5_D_5_N, δ in ppm).

Position	1	2	3
1	32.6	32.7	36.4
2	23.5	23.5	31.8
3	139.4	139.5	69.8
4	127.5	127.5	43.0
5	164.2	164.3	171.0
6	124.0	123.7	126.0
7	203.0	203.4	202.5
8	48.5	48.4	48.0
9	43.4	43.2	44.0
10	36.2	36.2	38.3
11	31.8	30.9	31.8
12	67.2	69.4	67.3
13	59.3	57.9	59.2
14	87.8	87.5	87.7
15	31.6	30.9	31.9
16	33.6	31.8	33.6
17	91.7	87.6	91.7
18	7.8	9.2	7.8
19	16.5	16.4	17.4
20	209.1	73.1	209.1
21	27.7	18.3	27.8

**Table 2:** ^1^H-NMR spectral data of compounds **1**–**3** (500 MHz, C_5_D_5_N, δ in ppm).

Position	1	2	3
1	1.21 (1H, m)	1.21 (1H, m)	1.17 (1H, ddd, *J* = 13.7, 13.4, 3.5 Hz)
	1.80 (1H, m)	1.81 (1H, m)	1.86 (1H, m)
2	2.03 (1H, m)	2.08 (1H, m)	1.80 (1H, m)
	2.15(1H, m)	2.14 (1H, m)	2.18 (1H, m)
3	6.16 (1H, m)	6.16 (1H, m)	3.89 (1H, m)
4	6.09 (1H, dd, *J* = 9.8, 1.9 Hz)	6.09 (1H, br d, 9.8 Hz)	2.60 (1H, m)
			2.75 (1H, m)
6	5.80 (1H, s)	5.80 (1H, s)	5.87 (1H, s)
8	2.82 (1H, d, *J* = 12.9 Hz)	2.82 (1H, d, *J* = 12.9 Hz)	2.71 (1H, d, *J* = 12.9 Hz)
9	1.83 (1H, ddd, *J* = 12.9, 9.9, 3.0 Hz)	1.83 (1H, ddd, *J* = 12.9, 9.9, 3.0 Hz)	1.78 (1H, m)
11	2.07 (1H, m)	2.04 (1H, m)	1.99 (1H, m)
	2.12 (1H, m)	2.10 (1H, m)	2.06 (1H, m)
12	3.78 (1H, dd, *J* = 11.4, 4.5 Hz)	3.78 (1H, dd, *J* = 11.4, 4.5 Hz)	3.79 (1H, m)
15	1.72 (1H, m)	1.75 (1H, m)	1.75 (1H, m)
	1.90 (1H, m)	2.06 (1H, m)	1.92 (1H, m)
16	2.10 (1H, m)	1.92 (1H, m)	2.11 (1H, m)
	3.42 (1H, m)	2.08 (1H, m)	3.43 (1H, m)
18	1.70 (3H, s)	1.70 (3H, s)	1.69 (3H, s)
19	1.01 (3H, s)	1.00 (3H, s)	1.12 (3H, s)
20		4.28 (1H, m)	
21	2.62 (3H, s)	1.60 (3H, d, *J* = 6.4 Hz)	2.63 (3H, s)
3-OH			6.62 (1H, br s)
12-OH	6.41 (1H, br s)	6.96 (1H, br s)	6.44 (1H, br s)
14-OH	6.04 (1H, s)	6.25 (1H, s)	5.88 (1H, s)
17-OH	5.18 (1H, s)	6.53 (1H, s)	5.23 (1H, s)
